# Supplementary material for: The Functional Significance of High Cysteine Content in Eye Lens γ-Crystallins
Source: Biomolecules. 2024 May 17;14(5):594. doi: 10.3390/biom14050594 (PMC11118217; doi:10.3390/biom14050594)
Supplement: Supplementary file 1 [file biomolecules-14-00594-s001.zip › crystallin_cysteines_SI.pdf]

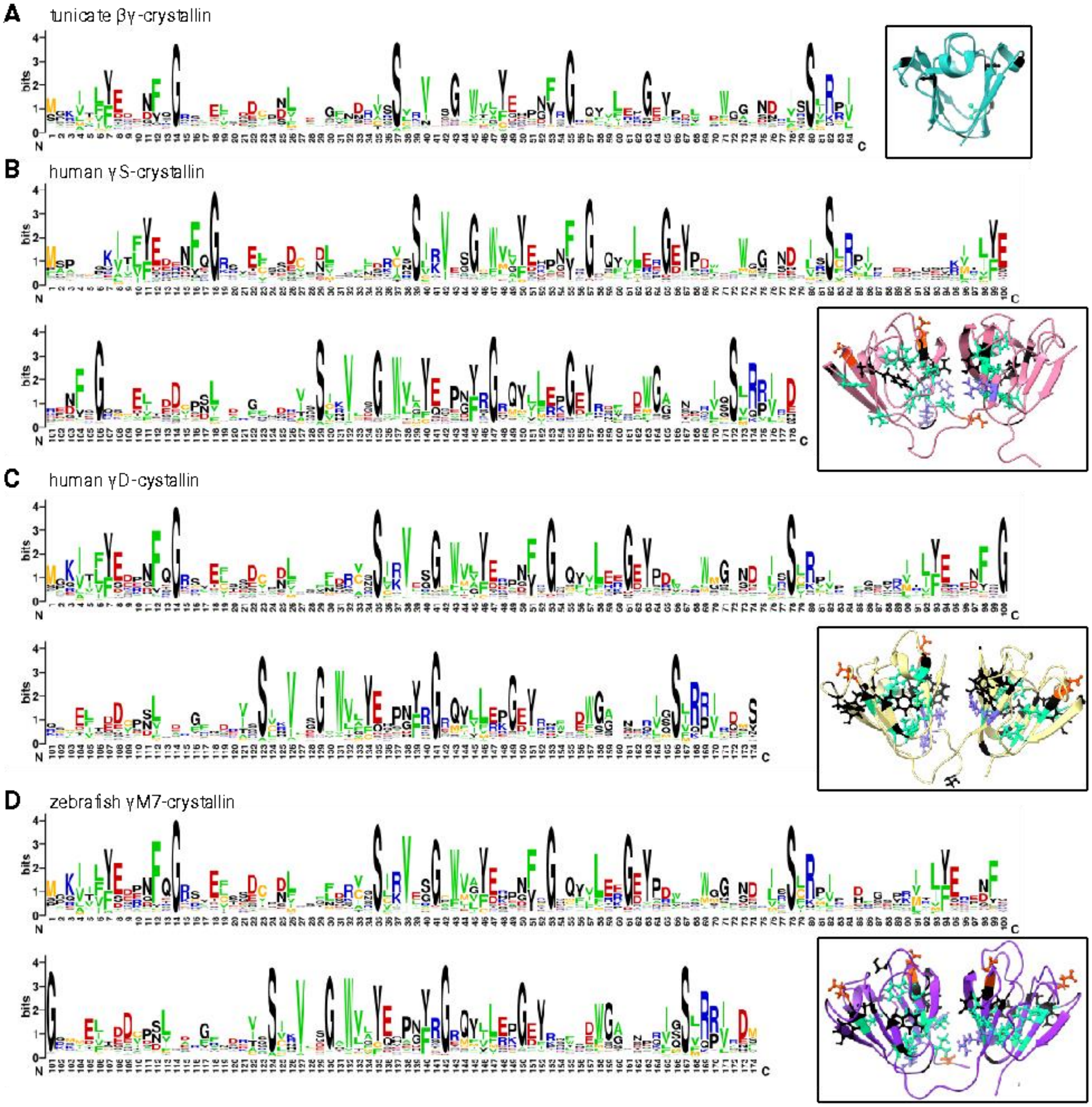

Figure S1. Multiple sequence alignment. The Deep MSA tool was used to identify conserved residues starting with **A** tunicate βγ-crystallin **B** human γS-crystallin **C** human γD-crystallin and **D** zebrafish γM7-crystallin. In all cases, the most common conserved residues are serine and glycine, along with the tryptophans commonly found in the hydrophobic core of each Greek key domain, the tyrosines associated with the tyrosine corners, and charged residues on the surface. Insets show the structure of each protein with strongly conserved residues highlighted. Cysteine and methionine are generally not among the more strongly conserved residues.

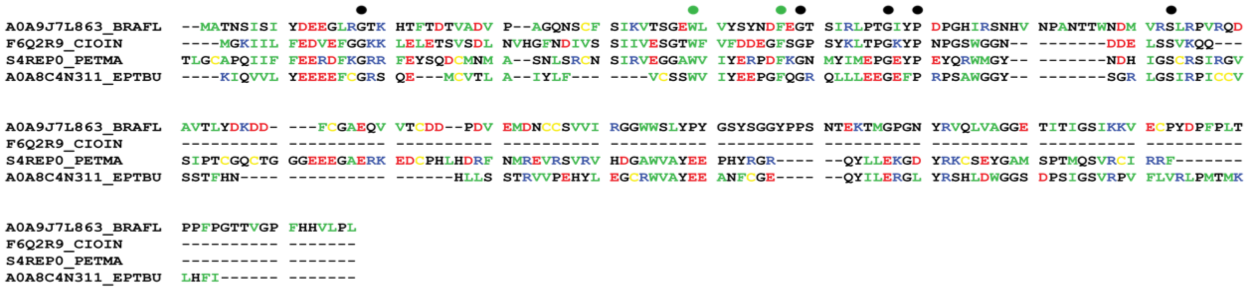

Figure S2. Sequence alignment for γ-crystallin Cluster 1 (βγ-crystallins).

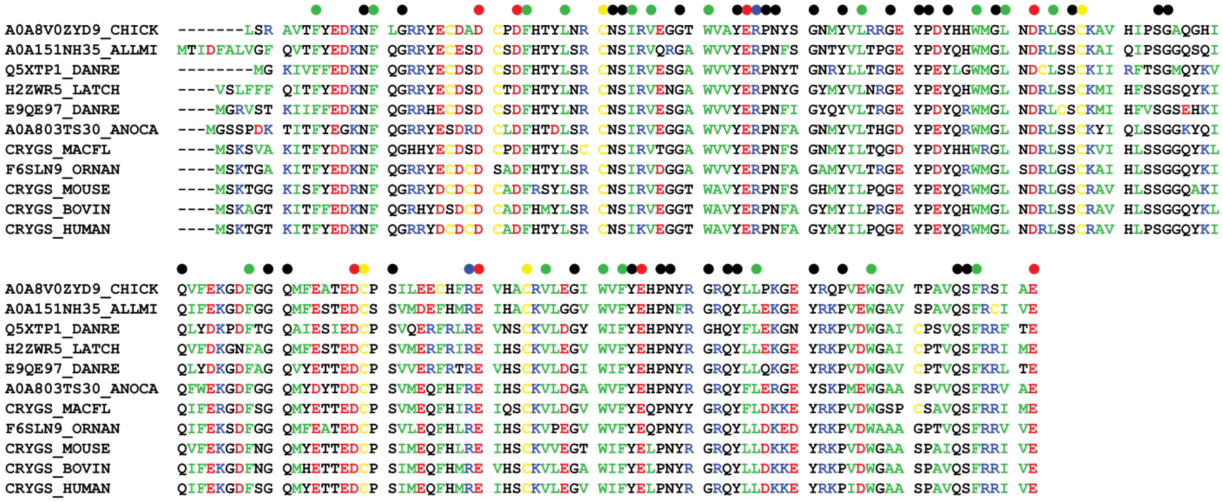

Figure S3. Sequence alignment for γ-crystallin Cluster 2 (γS-like crystallins).

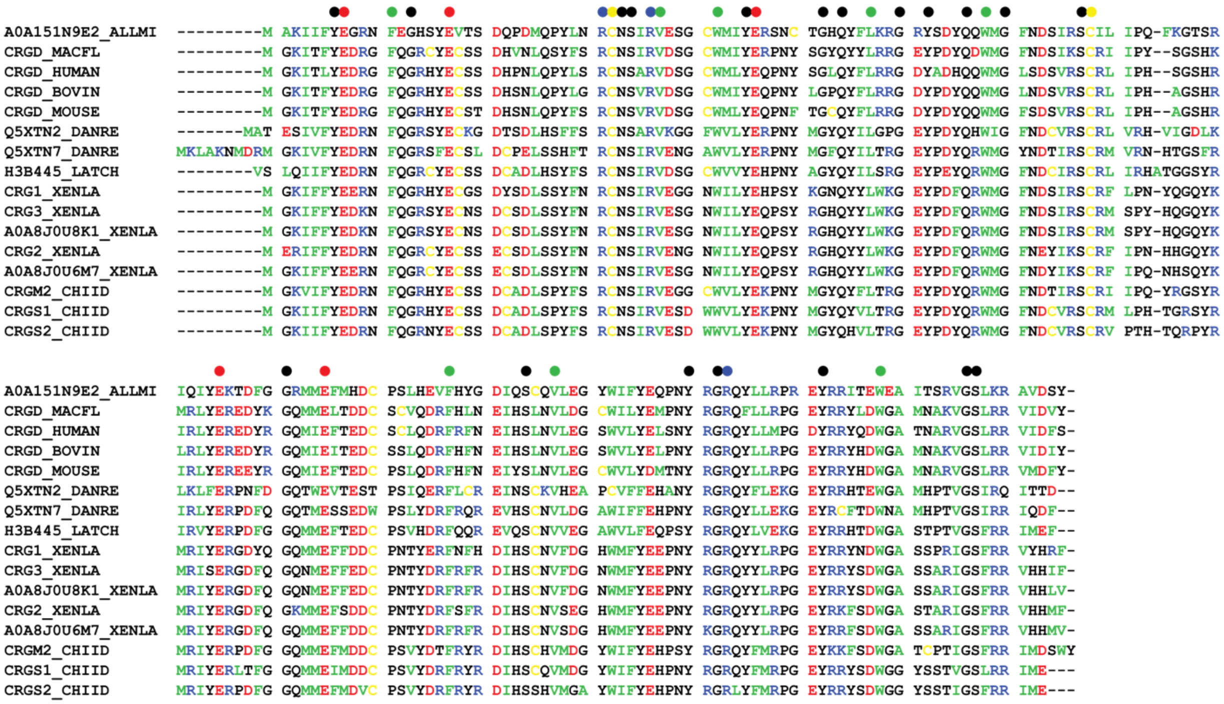

Figure S4. Sequence alignment for γ-crystallin Cluster 3 (γD-like crystallins.)

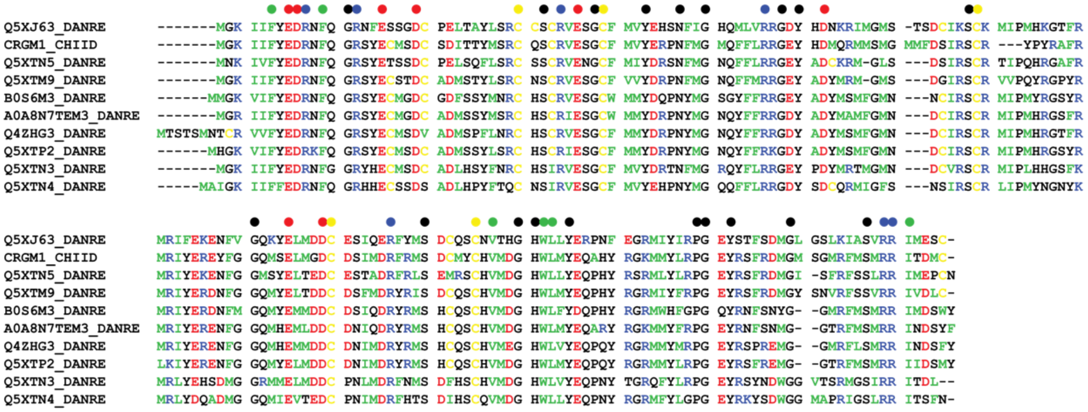

Figure S5. Sequence alignment for γ-crystallin Cluster 4 (γM-crystallins).

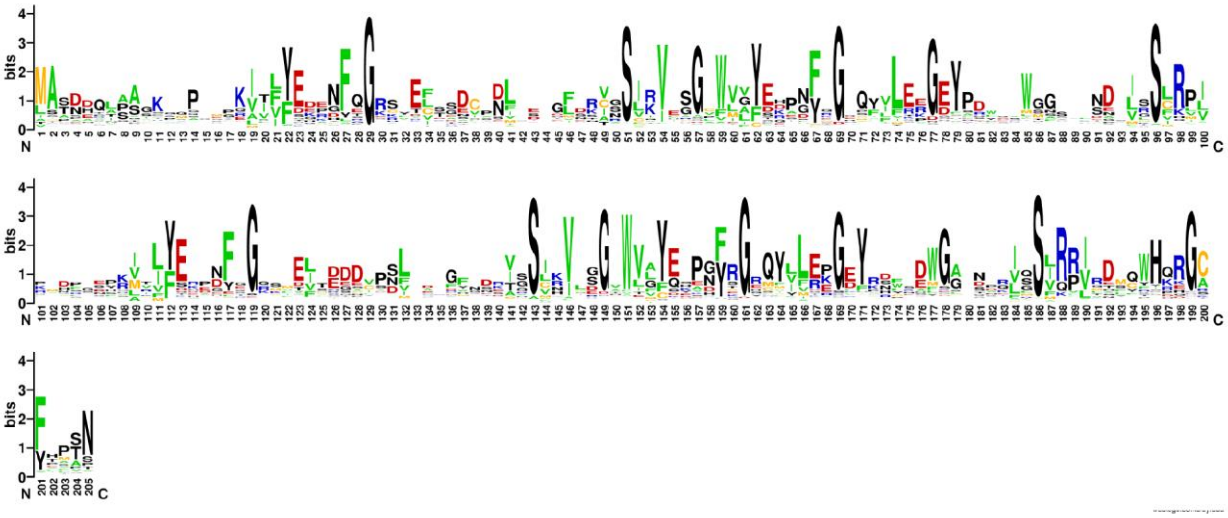

Figure S6. Multiple sequence alignment. The Deep MSA tool was used to identify conserved residues starting with human β2-crystallin. As for the γ-crystallins, the most common conserved residues are mostly serine, glycine, and aromatic residues, and not cysteine or methionine.

Table S1: Cysteine and methionine content of β-crystallins in representative vertebrates.

organism	protein	UniProt ID	method	length	Cys (no./%)	Met (no./%)
Mus musculus	βA1-crystallin	CRBA1_MOUSE	x	198	8/4.0	4/2.0
Ornithorhynchus anatinus	βA1-crystallin	F7BTY4_ORNAN	x	204	10/4.9	5/2.5
Danio rerio	βA1-crystallin	Q567D9_DANRE	x	199	11/5.5	8/4.0
Danio rerio	βA1-crystallin	Q6DH14_DANRE	x	196	7/3.6	9/4.6
Danio rerio	βA1b-crystallin	Q6DGY4_DANRE	x	198	12/6.0	12/6.0
Danio rerio	βA1c-crystallin	B5M4A7_DANRE	x	206	10/4.9	8/3.9
Latimeria chalumnae	βA1-crystallin	H3AXI2_LATCH	x	199	10/5.0	12/6.0
Latimeria chalumnae	βA1-crystallin-like	H2ZY80_LATCH	x	213	9/4.2	13/6.1
Anolis carolinensis	βA1-crystallin	H9GJE8_ANOCA	x	215	4/1.9	7/3.3
Gallus gallus	βA2-crystallin	CRBA2_CHICK	x	196	3/1.5	4/2.0
Alligator mississippiensis	βA2-crystallin	A0A151NRA2_ALLMI	x	208	4/1.9	3/1.4
Latimeria chalumnae	βA2-crystallin	H3B2W8_LATCH	x	213	7/3.3	6/2.8
Danio rerio	βA2-crystallin	Q6DGY2_DANRE	x	197	8/4.1	7/3.6
Danio rerio	βA2-crystallin	Q6IQU2_DANRE	x	197	8/4.1	9/4.6
Macropus fuliginosis	βA2-crystallin	CRBA2_MACFL	x	197	4/2.0	2/1.0
Ornithorhyncus anatinus	βA2-crystallin	A0A6I8NZG8_ORNAN	x	241	4/1.6	4/1.6
Bos taurus	βA2-crystallin	CRBA2_BOVIN	x	197	4/2.0	1/0.5
Mus musculus	βA2-crystallin	CRBA2_MOUSE	x	197	7/3.6	1/0.5
Homo sapiens	βA2-crystallin	CRBA2_HUMAN	x	197	6/3.0	1/0.5

Gallus gallus	βA3-crystallin	CRBA1_CHICK	x	215	7/3.3	7/3.3
Alligator mississippiensis	βA3-crystallin	A0A151N992_ALLMI	x	215	6/2.8	6/2.8
Homo sapiens	βA3-crystallin	CRBA1_HUMAN	x	215	5/2.3	6/2.8
Bos taurus	βA3-crystallin	CRBA1_BOVIN	x	215	8/3.7	6/2.8
Alligator mississippiensis	βA4-crystallin	A0A151MUM2_ALLMI	x	260	14/5.4	3/1.2
Gallus gallus	βA4-crystallin	CRBA4_CHICK	x	196	7/3.6	1/0.5
Homo sapiens	βA4-crystallin	CRBA4_HUMAN	x	196	5/2.5	2/1.0
Mus musculus	βA4-crystallin	CRBA4_MOUSE	x	196	4/2.0	2/1.0
Latimeria chalumnae	βA4-crystallin	H3ASM1_LATCH	x	205	9/4.4	7/3.4
Danio rerio	βA4-crystallin	Q52JI7_DANRE	x	196	11/5.6	5/2.6
Danio rerio	βB1-crystallin	A7E2K5_DANRE	x	221	9/4.1	15/6.8
Danio rerio	βB1-crystallin	E7F8M1_DANRE	x	219	9/4.1	16/7.3
Danio rerio	βB1-crystallin-like	A7E2I4_DANRE	x	208	8/3.8	12/5.8
Danio rerio	βB1-crystallin	Q6DGZ8_DANRE	x	232	9/3.9	12/5.2
Latimeria chalumnae	βB1-crystallin	H3A1B8_LATCH	x	219	10/4.6	14/6.4
Latimeria chalumnae	βB1-crystallin	H3AWX7_LATCH	x	211	10/4.7	10/4.7
Latimeria chalumnae	βB1-crystallin	H3AS10_LATCH	x	254	11/4.3	9/3.5
Gallus gallus	βB1-crystallin	CRBB1_CHICK	x	238	5/2.1	8/3.4
Alligator mississippiensis	βB1-crystallin	A0A151MUJ3_ALLMI	x	238	3/1.3	6/2.5
Bos taurus	βB1-crystallin	CRBB1_BOVIN	x	253	4/1.6	5/2.0
Mus musculus	βB1-crystallin	CRBB_MOUSE	x	250	4/1.6	5/2.0

Homo sapiens	βB1-crystallin	CRBB1_HUMAN	x	252	1/0.4	4/1.5
Latimeria chalumnae	βB2-crystallin	H3AY34_LATCH	x	205	4/2.0	1/0.5
Gallus gallus	βB2-crystallin	BRBB2_CHICK	x	219	3/1.4	2/0.9
Alligator mississippiensis	βB2-crystallin	A0A151NSD4_ALLMI	x	204	3/1.5	1/0.5
Macropus fuliginosis	βB2-crystallin	B2ZF66_MACFL	x	205	1/0.5	2/1.0
Ornithorhyncus anatinus	βB2-crystallin	F7A5Y5_ORNAN	x	187	3/1.6	1/0.5
Danio rerio	βB2-crystallin	Q52JI4_DANRE	x	208	6/2.9	1/0.5
Homo sapiens	βB2-crystallin	CRBB2_HUMAN	x	205	1/0.5	2/1.0
Bos taurus	βB2-crystallin	CRBB2_BOVIN	x	205	2/1.0	2/1.0
Mus musculus	βB2-crystallin	CRBB2_MOUSE	x	205	2/1.0	3/1.5
Danio rerio	βB3-crystallin	Q52JI3_DANRE	x	259	4/1.5	3/1.2
Latimeria chalumnae	βB3-crystallin	H3AXG5_LATCH	x	211	2/0.9	5/2.4
Gallus gallus	βB3-crystallin	CRBB3_CHICK	x	211	1/0.5	4/1.9
Ornithorhyncus anatinus	βB3-crystallin	F6Z4S4_ORNAN	x	211	3/1.4	1/0.5
Homo sapiens	βB3-crystallin	CRBB3_HUMAN	x	211	2/0.9	1/0.5
Mus musculus	βB3-crystallin	CRBB3_MOUSE	x	211	3/1.4	1/0.5
Bos taurus	βB3-crystallin	CRBB3_BOVIN	x	211	2/0.9	2/0.9

```
A0A9J7L863_BRAFL    ---------- ---------- ---------- ---------- ---------- --MATNSISI YDEEGLRGTK HTFTDTVADV PA--GQNSCF SIKVTSGEWL
A0A8C4N311_EPTBU    ---------- ---------- ---------- ---------- ---------- ----KIQVVL YEEEEFCGRS QEMCVTLA-- ---------- IYLFVCSSWV
F6Q2R9_CIOIN        ---------- ---------- ---------- ---------- ---------- ----MGKIIL FEDVEFGGKK LELETSVSDL NVHGFNDIVS SIIVESGTWF
S4REP0_PETMA        ---------- ---------- ---------- ---------- ---------- TLGCAPQIIF FEERDFKGRR FEYSQDCMNM AS--NLSRCN SIRVEGGAWV
A0A6I8NZG8_ORNAN    MRLLGRGEGS VVAGEHEDES EMDKGSLVSG TQAQEQREQE HISRMSSLSP QDSGRARLTL WEEEGFQGRR CELIGDCPSI RDRSGFRRVR SVKVESGAWV
CRBA2_MACFL         ---------- ---------- ---------- ---------- ----MSGTLS QGSSPARLTL WEEENFQGRR CELMSDCSSI RELSGFRRVR SVKVESGAWV
CRBA2_BOVIN         ---------- ---------- ---------- ---------- ----MSSAPA QGPAPASLTL WDEEDFQGRR CRLLSDCANI GERGGLRRVR SVKVENGAWV
CRBA2_MOUSE         ---------- ---------- ---------- ---------- ----MSSAPA PGSAPVCLTL WDEEDFQGRR CRLLSDCANV CERGALRRVR SVKVENGAWV
CRBA2_HUMAN         ---------- ---------- ---------- ---------- ----MSSAPA PGPAPASLTL WDEEDFQGRR CRLLSDCANV CERGGLPRVR SVKVENGVWV
H3B2W8_LATCH        ---------- ---------- --WPAFLLIS -----EVEKR SDFKMTTQQI ETMGQWKITV WEEENFQGKR CEFMMECPNI MER-GFHKIR SVKVECGPWV
CRBA2_CHICK         ---------- ---------- ---------- ---------- ----MTSEAM DTLGQYKITV WEEESFQGKR CEFLMECPSI MER-GFRKIR SIKVESGPWV
A0A151NRA2_ALLMI    ---------- ---------- -------MRG -----LRVCS KGIAMTTQAM DTLGQFKITV WEEENFQGKR CEFLVECPGI LER-GFRKIR SIKVENGPWV
Q6DGY2_DANRE        ---------- ---------- ---------- ---------- ---MNPQEQL EQRGKFRMTV FEEEHFQGKS CEFSFECQNI LER-DFRKIR SIKVENGPWV
Q6IQU2_DANRE        ---------- ---------- ---------- ---------- ---MNQREQM EQQCQWRITV WEEENFQGKR CEFRLECPNI LDR-DFQKIR SIKVDNGPWV

A0A9J7L863_BRAFL    VYSYNDFEGT SIRLPTGIYP DPGHIRSNHV NPANTTWNDM VRSLRPVRQ- ----DAVTLY DKDDFCGAE- ---QVVTCDD PDVE-----M DNCCSVVIRG
A0A8C4N311_EPTBU    IYEEPGFQGR QLLLEEGEFP RPSAWGGYSG R--------- LGSIRPICCV SSTFHNHL-- ---------- ---------- ------LSST RVVPEHYLEG
F6Q2R9_CIOIN        VFDDEGFSGP SYKLTPGKYP NPGSWGGNDD E-----LSS- ---VKQQ--- ---------- ---------- ---------- ---------- ----------
S4REP0_PETMA        IYERPDFKGN MYIMEPGEYP EYQRWMGYND H--------- IGSCRSIRGV S-------IP TCGQCTGGGE EEGAERKEDC PHLH-DRFNM REVRSVRVHD
A0A6I8NZG8_ORNAN    GFEHPDFQGQ QFILERGDYP CWLAWSGSSG Y-----HTDQ LLSFRLLQCA NHSDSRVTLF EGENFQGG-- --KFEVNDDY PSLPAMGWTS SVVGSLMVGS
CRBA2_MACFL         GFEYPDFQGQ QFILEKGDYP RSTAWSGSSG Y-----RTDQ LLSFRPLLCA NHSDSRVTLY EGENFQGC-- --KFELSDDY PSLPAMGWAS KDVGSLKVTS
CRBA2_BOVIN         AFEYPDFQGQ QFILEKGDYP RWSAWSGSAG H-----HSDQ LLSFRPVLCA NHSDSRVTLF EGENFQGC-- --KFELNDDY PSLPSMGWAS KDVGSLKVSS
CRBA2_MOUSE         AFEYPDFQGQ QFILEKGDYP CWSAWSGSSG H-----HSNQ LLSFRPVLCA NHSDSRVTLF EGENFQGC-- --KFELSDDY PSLPSMGWTS KDVGSLKVSS
CRBA2_HUMAN         AFEYPDFQGQ QFILEKGDYP RWSAWSGSSS H-----NSNQ LLSFRPVLCA NHNDSRVTLF EGDNFQGC-- --KFDLVDDY PSLPSMGWAS KDVGSLKVSS
H3B2W8_LATCH        GFEYPEFQGQ QFILEKGDYP RWEAWSGNSG Y-----RTEH LLSFRPVRCA NHSDSKVTLY ECENFQGR-- --KFELCDDY PSLQAMGWCN KEVASVKVNS
CRBA2_CHICK         GFEYPEYQGQ QFILEKGDYP RWEAWSGNSG Y-----RTEH LLSFRPVKCA NHNDSKAILY EAENFQGH-- --KFELSDDY PSLQAMGWGN KEVASIKVNA
A0A151NRA2_ALLMI    GFEYPEFQGQ QFILEKGDYP RWEAWSGSSG Y-----RTEH LLSFRPIKCA NHNDSKVTLY EGENFQGR-- --KFELSDDY PSLQAMGWGT KEVASIKVNS
Q6DGY2_DANRE        GYEYPEFQGR QFILEKGDYP CYQAWSGNGS Y-----RTEH MLSFRPIKCA NHSDSKITLY ECEDFMGR-- --KFEMCEDY PSIQAMGWCS KEVPSIKVNS
Q6IQU2_DANRE        GYEYPEYQGQ QFILEKGDYP CYQAWSGNSS Y-----RTEH MLSFRPIKCA NHSDSKITMY ECEDMMGR-- --KFEMCDDY PSLMAMGWCS KEVPSIKVNS

A0A9J7L863_BRAFL    GWWSLYPYGS YSGGYPPSNT EKTMGPGNYR VQLVAGGETI T--IGSIKKV ECPYDPFPLT PPFPGTTVGP FHHVLPL
A0A8C4N311_EPTBU    CRWVAYEEAN FCGEQYIL-- ER----GLYR SHLDWGGSDP S--IGSVRPV FLVRLPMTMK LHFI------ -------
F6Q2R9_CIOIN        ---------- ---------- ---------- ---------- ---------- ---------- ---------- -------
S4REP0_PETMA        GAWVAYEEPH YRGRQYLL-- EK----GDYR KCSEYGAMSP T--MQSVRCI RRF------- ---------- -------
A0A6I8NZG8_ORNAN    GAWVAYQYPG YRGFQYVL-- ERDRHGGEFR KYSELGTQAA TGQIQSIRRI QQ-------- ---------- -------
CRBA2_MACFL         GAWVGYQYPG FRGYQYVL-- EQDRHSGEFR KYSEFGTQAH TNQLQSIRRV QH-------- ---------- -------
CRBA2_BOVIN         GAWVAYQYPG YRGYQYVL-- ERDHHSGEFR NYSEFGTQAH TGQLQSIRRV QH-------- ---------- -------
CRBA2_MOUSE         GAWVAYQYPG YRGYQYVL-- ERDRHSGEFR TYSDFGTQAH TGQLQSIRRV QH-------- ---------- -------
CRBA2_HUMAN         GAWVAYQYPG YRGYQYVL-- ERDRHSGEFC TYGELGTQAH TGQLQSIRRV QH-------- ---------- -------
H3B2W8_LATCH        GAWVAYQYPG YRGYQYVL-- ERDRHSGEYK NYNEYSTQAH TNQIQSIRRI QH-------- ---------- -------
CRBA2_CHICK         GAWVAYQYPG YRGYQYVL-- ERDRQNGEFK KYSEYSSQAH TNQIQSIRRI QH-------- ---------- -------
A0A151NRA2_ALLMI    GAWVAYQYPG YRGYQYIL-- ERDKQNGEFK KYIEYSSQAH TNQIQSIRRV QH-------- ---------- -------
Q6DGY2_DANRE        GAWVGYQFPG YRGYQYIF-- ERDRHEGEYR CYNDFGTQAH TNQIQSMRRI QQ-------- ---------- -------
Q6IQU2_DANRE        GAWVGYQFPG YRGYQYIF-- ERDRRQGEYR KYYEFGTQAH SNQIQSMRRI QH-------- ---------- -------
```

Figure S7. Sequence alignment for β-crystallin Cluster 1 (βγ and βA2-crystallins).

```
Q6DGY4_DANRE      ---------- -------MAL TNPMSMPMGP WKITVYDQEH FQGRRCEFTA CCQNIMEYGM ETVRSLKVEC GAWVGFEHST FNGQQFILEK GDYPCWEAWS
H9GJE8_ANOCA      MEELSAFSER DFTSVAKMAQ TNPQQAQFGP WKITIYDQEN FQGKRMEFAA SCPSIMECSF DNIRSFKVES GAWFGYEHTN YCGQQFVLER GEYPRWEAWS
Q6DH14_DANRE      ---------- -------MAL TN--PMPLGP WKLTVYDQEN FQGKRMEFTA ACQNIMECGM DNVRSLKVEC GAWAGYEHSS FCGQQFILER GDYPRWESWS
A0A151N992_ALLMI  MEDPAAQTEL DSTPVARMAQ TNPLPVPTGP WKITLYDQEN FQGKRMEFTT SCANIMECSF DNIRSLKVDC GAWIGYEHTG FCGQQFTLER GEYPRWDAWS
CRBA1_CHICK       MGEAAVPPEL DTFPAAKMAQ TNPLPVPMGP WKITVYDQEN FQGKRMEFTS ACPNIMECGF DNIRSLKVEC GAWVGYEHTG FCGQQFILER GEYPRWDAWS
H3AXI2_LATCH      ---------- ------KMAQ TNPMPMPMGP WKITVYDQEC FQGKRMEFTS SCPNIMECGF DNVRSIKVEC GAWVGYEHSS FCGQQFILER GEYPRWDAWS
F7BTY4_ORNAN      ---------- -TLSPARMTQ TNPLPGPLGP WKVTIYDQEN FQGKRMEFTA SCPNVTECGF DNIRSLKVEC GAWIGFEHTS FCGQQFVLER GDYPCWEAWS
CRBA1_MOUSE       ---------- -------MLY LVLFLVPFNS IQITIYDQEN FQGKRMEFTS SCPNVSERNF DNVRSLKVEC GAWIGYEHTS FCGQQFILER GEYPRWDAWS
CRBA1_HUMAN       METQAEQQEL ETLPTTKMAQ TNPTPGSLGP WKITIYDQEN FQGKRMEFTS SCPNVSERSF DNVRSLKVES GAWIGYEHTS FCGQQFILER GEYPRWDAWS
CRBA1_BOVIN       METQTVQQEL ESLPTTKMAQ TNPMPGSVGP WKITIYDQEN FQGKRMEFTS SCPNVSERNF DNVRSLKVEC GAWVGYEHTS FCGQQFVLER GEYPRWDAWS
H2ZY80_LATCH      MQKESASKQE MGMGFGMR-- MGMGMGFSPF WKIVVWEQEY FQGKYQEFTS ECCNIMDCGF DNIRSVRVES GAWVGYEHHD FQGQQFILER GEYPRWEGWS
Q567D9_DANRE      ---------- ----MQPM-- ISSGMGMAPF FRVTVFEQEH FQGKCQEFTN ECCNIQCCGF DNIRSIRVES GAWVGYEHHD FQGQQFILER GEYPHWDAYS
B5M4A7_DANRE      MNRV------ -TAIQTNP-- SASGIDMAPC FKVTVFEQEH FQGKCQELTS ECRNIQERGF DNIRSIRVES GAWVGYEHHD FQGQQFVLEK GEYPQWDAYS
H3ASM1_LATCH      ---------- ---------- ------FSGH WKIIVWDEEC FQGRRHEFTA ECCNIMEFGF ETVRSFRIES GAWVGYEHAA YMGQQFVLER GEYPRWDAWS
Q52JI7_DANRE      ---------- -------M-- THHCTKFSGH WKIIVYDEEC FQGRHHEFTS ECCNVMEFGF ESVRSLRVES GAWVGYEHAS YQGHQFVLER GEYPQCDSFG
CRBA4_CHICK       ---------- -------M-- IQRRRPSSGL WKIVVWDEPF FQGKRHEFTT DCYSTPERGF STVRSFRIES GAWVGFEHCG FQGQQFVLER GEYPCWEAWS
A0A151MUM2_ALLMI  ---------- -------M-- SQHCTKFSGL WKIVVWDEKN FQGRRHEFTS ECYNITELSF DTVRSFKIES GAWVGFEHGG FQGQQFVLER GEYPSWEAWS
CRBA4_HUMAN       ---------- -------M-- TLQCTKSAGP WKMVVWDEDG FQGRRHEFTA ECPSVLELGF ETVRSLKVLS GAWVGFEHAG FQGQQYILER GEYPSWDAWG
CRBA4_MOUSE       ---------- -------M-- TLQCTKSAGH WRMVVWDEEG FQGRRHEFTA ECPSVLELGF ETVRSLKVLS GAWVGFEHAG FQGQQYVLER GDYPGWDAWG

Q6DGY4_DANRE      GNNAYRIERL ISFRPIYSAM HSDSRMMLFD CENMTGKQWE VCNDYPSLQG MGWCNNEVRS IQVMSGAFVC YQYPGYRGYQ YLMECDCHGG EYRHYREYGC
H9GJE8_ANOCA      GSNAYHVERL MSFRPINSAN HKESKITLFE KENFMGHQWD LQDDYPSLQA MGWGSNEVGS MKVHSGAWVC YQYPGYRGYQ YILEFDHHGG DYKHWREWGS
Q6DH14_DANRE      GSNAYHIERL MSFRPICSAN HKESKITVFE RENFIGHQWE MTDDYPSLQA MGWPSNEIGS MQVQSGAWVC YQYPGYRGYQ YIMECDHHGG EYKHYREWGS
A0A151N992_ALLMI  GSNAYHVERL MSFRPIGSAN HKESKITLFE KENFLGRQWE IGDDYPSLQA MGWANNEVGS MKVLCGAWAC YQYPGYRGYQ YILESDHHGG DYKHWREWGS
CRBA1_CHICK       GSNAYHIERL MSFRPVCSAN HKESKITVYE KDNFIGRQWE ISDDYPSLQA MGWANNEVGS MKIPCGAWVC YQYPGYRGYQ YVLEADHHGG DYKHWREWGS
H3AXI2_LATCH      GSNSYHIERM MSFRPVCSAS HKESKIVIFE KENFIGRQWE MCDDYPSLQA MGWCNNEVGS MKIQSGAWVC YQFPGYRGYQ YIMECDHHGG EYKHWREWGS
F7BTY4_ORNAN      GSNAYHIERL TSFRPICSAN HKESKITIFE KQNFIGRQWE LCDDYPSLQA MGWPNNEVGS MKIQCGAWVC YQYPGYRGYQ YILECDHHGG DYKHWREWGS
CRBA1_MOUSE       GSNAYHIERL MSFRPICSAN HKESKITIFE KENFIGRQWE ICDDYPSLQA MGWFNNEVGS MKIQCGAWVC YQYPGYRGYQ YILECDHHGG DYKHWPEWGS
CRBA1_HUMAN       GSNAYHIERL MSFRPICSAN HKESKMTIFE KENFIGRQWE ISDDYPSLQA MGWFNNEVGS MKIQSGAWVC YQYPGYRGYQ YILECDHHGG DYKHWREWGS
CRBA1_BOVIN       GSNAYHIERL MSFRPICSAN HKESKITIFE KENFIGRQWE ICDDYPSLQA MGWPNNEVGS MKIQCGAWVC YQYPGYRGYQ YILECDHHGG DYKHWREWGS
H2ZY80_LATCH      GSNSYHVERL MSFRPVYCAS HHGSRMTIYE RENFMGRCAE LCDDYPSLQA MGWCNQEVGS MHVQSGAWVC YQYPGYRGYQ YIMECERHSG DYQRWWNWGS
Q567D9_DANRE      GNLSYHVERL MSFRPIYCAS HQSSRMTIFE RENFLGRSAE LCDDYPSLQA MGWCRPEVGS MHVQCGAFVC YEFPGYKGRQ YIMECERHSG DFQHWRNWGS
B5M4A7_DANRE      GNLGYHVERM MSLRPICSAV HQDSRMTIFE KENFMGRSVE LCDDYPSLQA MGWCDKQVGS MHVQCGAFVC YEYPGYRGQQ YIMECDKHSG DYQHWKDWGS
H3ASM1_LATCH      GSNAYHVERM TSFRPIACAN HRDCRMSIYE QENFLGRKGE VSDDYPSLQA MGWCNNEVGS FRIHSGAWVC YQYPGYRGYQ YIMECDRHSG EYKHWREWGS
Q52JI7_DANRE      GSNAYHIERM TSFRPISCAN HRECRMTIYE RENYLGRKGE LSDDYPSLQA MGWCNNEVGS LRVQSGAFVC YQFPGYRGYQ YIMECDRHCG EYKQFREFGS
CRBA4_CHICK       GSNAYHVDRM SSFRPITCAV HGRSQLLLFE QENFQGRRGE LSDDCPSLPA LGWGGSAVGS FLVCSGAWVC SQYPGYRGFQ YLLESDTHAG EYKHVREWGS
A0A151MUM2_ALLMI  GSNAYHVERM NSFRPIACAN HRDSKITIYE RENFLGRKGD LSDDYPSLPA MGWNSKEVGS FRVHSGAWVC FQYPGYRGFQ YILECDCHGG DYKHFWELGS
CRBA4_HUMAN       GNTAYPAERL TSFRPAACAN HRDSRLTIFE QENFLGKKGE LSDDYPSLQA MGWEGNEVGS FHVHSGAWVC SQFPGYRGFQ YVLECDHHSG DYKHFREWGS
CRBA4_MOUSE       GNTAYPAERL TSFRPVACAN HRDSRLTIFE QENFLGRKGE LNDDYPSLQA MGWDGTEVGS FHVQSGAWVC SQFPGYRGFQ YILESDHHSG DYKHFREWGS

Q6DGY4_DANRE      HAQTPQIQSI RRIQH----- ---------- ---------- ---------- ---------- ---------- ---------
H9GJE8_ANOCA      HAQTAQIQSI RRVQQ----- ---------- ---------- ---------- ---------- ---------- ---------
Q6DH14_DANRE      HAQTFQVQSL RRVQQ----- ---------- ---------- ---------- ---------- ---------- ---------
A0A151N992_ALLMI  HAQTFQIQSL RRIQQ----- ---------- ---------- ---------- ---------- ---------- ---------
CRBA1_CHICK       HAQTSQIQSI RRIQQ----- ---------- ---------- ---------- ---------- ---------- ---------
H3AXI2_LATCH      HAQTFQVQSI RRIQQ----- ---------- ---------- ---------- ---------- ---------- ---------
F7BTY4_ORNAN      HAQTFQIQSM RRIQQ----- ---------- ---------- ---------- ---------- ---------- ---------
CRBA1_MOUSE       HAQTSQIQSI RRIQQ----- ---------- ---------- ---------- ---------- ---------- ---------
CRBA1_HUMAN       HAQTSQIQSI RRIQQ----- ---------- ---------- ---------- ---------- ---------- ---------
CRBA1_BOVIN       HAQTSQIQSI RRIQQ----- ---------- ---------- ---------- ---------- ---------- ---------
H2ZY80_LATCH      HAQTPQIQSI RRIQH----- ---------- ---------- ---------- ---------- ---------- ---------
Q567D9_DANRE      HSQTPQIQSI RRIQH----- ---------- ---------- ---------- ---------- ---------- ---------
B5M4A7_DANRE      HCQTPQIQSI RRIQH----- ---------- ---------- ---------- ---------- ---------- ---------
H3ASM1_LATCH      HAQTFQIQSI RRIQQFHHHE QGMYCRKQSQ Q--------- ---------- ---------- ---------- ---------
Q52JI7_DANRE      HSQTPQIQSI RRIQQ----- ---------- ---------- ---------- ---------- ---------- ---------
CRBA4_CHICK       HAQTGQVQSI RRVQQ----- ---------- ---------- ---------- ---------- ---------- ---------
A0A151MUM2_ALLMI  HAQTCQVQSI RRIQHLACSA FPHCCSQLLM TDLRSVHTQS PCGAGQDGGH RCSWAQGCRE QKSLARARCA LLLSTNRLR
CRBA4_HUMAN       HAPTFQVQSI RRIQQ----- ---------- ---------- ---------- ---------- ---------- ---------
CRBA4_MOUSE       HAHTFQVQSV RRIQQ----- ---------- ---------- ---------- ---------- ---------- ---------
```

Figure S8. Sequence alignment for β-crystallin Cluster 2 (βA4-crystallins).

```
Q52JI3_DANRE        ---------- ---------- ---------- ---------- ------MSDQ QGVPDQQVAG K-SQGGAGAI YKVSLYEFEN FRGKKLELSA ECKDLIEKNL
F6Z4S4_ORNAN        ---------- ---------- ---------- ---------- ------MTEQ QSVPQQAAAG K-AHGGLGGS FKITVFELEN FQGKRYDLTR ECPNLTEDRL
CRBB3_HUMAN         ---------- ---------- ---------- ---------- ------MAEQ HGAPEQAAAG K-SHGDLGGS YKVILYELEN FQGKRCELSA ECPSLTDSLL
CRBB3_BOVIN         ---------- ---------- ---------- ---------- ------MAEQ HSTPEQAAAG K-SHGGLGGS YKVIVYEMEN FQGKRCELTA ECPNLTESLL
CRBB3_MOUSE         ---------- ---------- ---------- ---------- ------MAEQ HGAPEQAAAG K-SHGGLGGS YKVTVYELEN FQGKRCELSA ECPNLTDSLL
CRBB3_CHICK         ---------- ---------- ---------- ---------- ------MTEQ QSPPEQMVTG E-GAGERGGN YKITIYELEN FQGRRCELSE ELPNVVDKAL
H3AXG5_LATCH        ---------- ---------- ---------- ---------- ------MTEQ QSVPEQMAAG K-SHGGIEGN YKMIIYELEN FQGKKSELSG ECQNLSEKGL
H3AWX7_LATCH        ---------- ---------- ---------- ---------- -------PLA SGTKTKYKTN -KGTMLSVHF IQIIFYEYEN FQGRKMEFTS ECKNLCDRGF
A7E2I4_DANRE        ---------- ---------- ---------- ---------- ---MSHTAVQ GSMGSRFAT- ------GKSS HRIYLFEYEN FQGHMMELSG ECRNVCDKGL
H3A1B8_LATCH        ---------- ---------- ---------- --MAKQSRRN ----V--PAP V--DQRQTLS L-ALTCLSLL FQMTIFDQEN FQGRCMEFSG ECMNVCDRGF
A7E2K5_DANRE        ---------- ---------- ---------- --MSTGGEKS ----K--PAS QPDGKAAQTQ K-KTEMGMGN YKMYVFDQEN FQGRMIEISN ECMNVCEMGM
E7F8M1_DANRE        ---------- ---------- ---------- --MSSSGEK- ----K--TAS QTDGKGA-PS K-KSEMGMMS YRMYVFDQEN FQGRMIEINA ECMNVCDMGM
CRBB1_HUMAN         -MSQAAKASA SATVAVNPGP DTKGKGAPPA GTSPSPGTTL APTTVPIT-- --------SA K-AAELPPGN YRLVVFELEN FQGRRAEFSG ECSNLADRGF
CRBB1_BOVIN         MSQP--AAKA SATAAVNPGP DGKGKAGPPP GPAPGSGPAP APAPAPAQ-- ------PAPA A-KAELPPGS YKLVVFEQEN FQGRRVEFSG ECLNLGDRGF
CRBB1_MOUSE         -MSQ--AAKA SATTAVNPGP DGKGKGAPST GPAPAPGPTP VPASVPRP-- --------AA K-VGDLPPGS YRLIVFEQEN FQGRRVEFSG ECLNLGDRGF
Q6DGZ8_DANRE        -MSQTAKSA- ----TN-QGT DAKEKGA--- ---------- -------PAP AAT---SKAS KTGDPGFMGN YKIFLFDQEN FQGRMMEVQN ECMNVCERGM
H3AS10_LATCH        -MSQTTKAA- ----TSGTDA KEKSTSTPAP APAPAPAPAP APAPAPAPAP AATPAASKAT K-TGEPSVGS YRIIIFEQEN FQGRHMEFMN ECVNLCDRGF
CRBB1_CHICK         -MSETTKTA- ----APGQAA EDKEKA---- ---------- ----APAPAP SSDPTPVTNS K-GEEPSTEA FRIVIFEQEN FQGRQMEFTS ECLNLADCGF
A0A151MUJ3_ALLMI    -MSDTTKPA- ----APSQAV DDKDKG---- ---------- ----VPAPAP VADPIPVSNS K-GEDSALEG FRIVVFEQEN FQGKKMEFTA ECLNLGERGF

Q52JI3_DANRE        EKVGSIIVES GPWVGFEQKG FLGEQFVLEK GEYPRWSTWT NSQNSFCLLS IRPLRVDS-A DHKLHLFENC SFAGRKMEIV DDDIPSLWVH GFQDRVASAK
F6Z4S4_ORNAN        EKVGSIQVEQ GPWLGFECHD FRGERFVLEK GDYPRWDAWS NSHRSDSLLS LRPLHVDG-P DHKLHLFENP GFGGRKMEIV DDDVPSLWAH GFQDRVASVR
CRBB3_HUMAN         EKVGSIQVES GPWLAFESRA FRGEQFVLEK GDYPRWDAWS NSRDSDSLLS LRPLNIDS-P HHKLHLFENP AFSGRKMEIV DDDVPSLWAH GFQDRVASVR
CRBB3_BOVIN         EKVGSIQVES GPWLAFERRA FRGEQYVLEK GDYPRWDAWS NSHHSDSLLS LRPLHIDG-P DHKLHLFENP AFGGRKMEIV DDDVPSLWAH GFQDRVASVR
CRBB3_MOUSE         EKVGSIQVES GPWLAFERRA FRGEQFVLEK GDYPRWDAWS SSRRSDILLS LRPLHIDG-P DHKLHLFENP AFSGRKMEIV DDDVPSLWAH GFQDRVASIR
CRBB3_CHICK         EKVGSIQVES GPWLGFERQA FAGEQFVLEK GDYPRWDSWS NSHNSDSLMS LRPLQIDS-P DHKIHLFENA GYTGRKMEIV DDDVPSLWAH GFQDRVASVR
H3AXG5_LATCH        EKVGSVQVES GPWIGFERQA FGGEQFVLEK GDYPRWDSWS NSHNSDSLMS VRPLRIDS-S DHKIHLFENA GYNGRKMEIV DDDVPSLWAH GFQDRVASVK
H3AWX7_LATCH        DKVGSIRVDC GPWMSYEQQN FCGEMFMLEK GEYPRWDSWS NSYRTDRIMS FRPVRMDN-Q DHKICLYECV NFEGRKMEVC DEDIPTLWSY GFQDRVASIQ
A7E2I4_DANRE        KRVGSIRVEC GPWVGFEQQN MAGEMFMLER GDYPLWATWS NSYRCDHLMS VRPVRMNP-Q DHNICLYECM NFDGRKMEVS DEDIPSLWCH GFQDRVGSIQ
H3A1B8_LATCH        DRIRSIRIDC GPWVGYEQSN FCGEMFIMEK GEYPRWDCWT NSYRNEWMMS FRPVLMDP-E KHKICLYEMG EFKGRKMEIM DDDVPSLFAY GFTDRVGSVS
A7E2K5_DANRE        DRVRSLRVEC GPWVGWEQMN FCGEMYILEK GEYPRWDCWS NCHRNDYLLS FRPIRMDP-E KHKICLYEVG DFKGRKMEII DDDVPSMYTF GFTDRVGSIM
E7F8M1_DANRE        DRVRSLRVEC GPFVGFEQMN FCGEMYILEK GEYPRWDSWS NCQKNDYLLS FRPVRMDP-E KHKICLYEVG DYKGRKMEIM DDDVPSLFSY GFTDRVGSIM
CRBB1_HUMAN         DRVRSIIVSA GPWVAFEQSN FRGEMFILEK GEYPRWNTWS SSYRSDRLMS FRPIKMDA-Q EHKISLFEGA NFKGNTIEIQ GDDAPSLWVY GFSDRVGSVK
CRBB1_BOVIN         ERVRSIIVTS GPWVAFEQSN FRGEMFVLEK GEYPRWDTWS SSYRSDRLMS FRPIKMDA-Q EHKLCLFEGA NFKGNTMEIQ EDDVPSLWVY GFCDRVGSVR
CRBB1_MOUSE         DRVRSLIVVS GPWVAFEQSA FRGEMFVLEK GEYPRWDTWT SSYRSDRLMS FRPIRMDS-Q EHKICLFEGA NFKGNTMEIQ EDDVPSLWVY GFCDRVGSIT
Q6DGZ8_DANRE        DRVRSIIVEC GPFVAFEQTN FRGEMFILEK GEYPRWDTWS NSYRSDCLMS LRPFRMDP-M EHKICLFELS DFKGNKMEIQ EDDVPTLWAH GFCDRVGSVR
H3AS10_LATCH        DRVRSVIIEC GPWVAFEQSN FRGEMFILEK GEYPRWDTWS NSYRSDCFMS FRPIRMQDSQ EHKICLFECA DFQGNKMEIF EDDVPSLWAY GFCDRVGSVR
CRBB1_CHICK         DRVRSVIVSS GPWVAYEQAN MRGEMFILEK GEYPRWDTWS SSYRSDCFMS MRPIRMEA-E DHKISLYESA DFKGNKMDIQ EDDVPSLWAY GFCDRVGSVK
A0A151MUJ3_ALLMI    DRVRSVIINS GPWVAFEQAN LRGEMFILEK GEYPRWDTWS SSYRSDCFMS MRPIKMEA-E DHKITLFESA DFKGNKMEIQ EDDVPSLWAH GFCDRVGSVK

Q52JI3_DANRE        AVNGTWVGYM YPGYRGRQFV FEHGDYKHWN DWGATEPQLQ SVRRVRDMQW HKRGCFTVPT PDPAPKPNPN PNPNPNPNPN PNPNPNPAPN PAPAPAPPAP
F6Z4S4_ORNAN        ALNGTWVGYE YPGYRGRQFV FEKGEYGHWN EWGAGQPLVQ SVRRVRDQKW HRRGCYPSS- ---------- ---------- ---------- ----------
CRBB3_HUMAN         AINGTWVGYE FPGYRGRQYV FERGEYRHWN EWDASQPQLQ SVRRIRDQKW HKRGRFPSS- ---------- ---------- ---------- ----------
CRBB3_BOVIN         AINGTWVGYE FPGYRGRQYV FERGEYRHWN EWDANQPQLQ SVRRIRDQKW HKRGVFLSS- ---------- ---------- ---------- ----------
CRBB3_MOUSE         VINGTWVGYE FPGYRGRQYV FERGEFRHWN EWDANQPQLQ SVRRIRDQKW HKRGCFLSS- ---------- ---------- ---------- ----------
CRBB3_CHICK         ALNGTWVGYE YPGYRGRQHV FEKGEYRHWN EWDANQPLMQ SVRRVRDQQW HQRGSFENS- ---------- ---------- ---------- ----------
H3AXG5_LATCH        AVNGTWVGYE YPGYRGHQYI FEKGEYRHWN EWAANQPQIQ SIRRIRDMQW HKRGCFASS- ---------- ---------- ---------- ----------
H3AWX7_LATCH        VMGGTWVGYQ YPGYRGYQFV FECGPYKHWN EWGACQPQIQ SIRRVKDMQW YRRGCFEFTN ---------- ---------- ---------- ----------
A7E2I4_DANRE        VNGGTWVGYQ YPGYRGYQYL FEFGSYKHWN EWGANHPQIQ SVRRVRDMQT HHRGCFELA- ---------- ---------- ---------- ----------
H3A1B8_LATCH        VPCGTWVGYQ YPGYRGYQYL FEKGEYHHWN DWNARHPQMQ SMRRIRDMQW HQHGCYTYAS K--------- ---------- ---------- ----------
A7E2K5_DANRE        VSCGTWVGYQ YPGYRGSQYL LEKGDYRHFN EYGARHPQFQ SVRRIRDMQW HPDGCYTMAT K--------- ---------- ---------- ----------
E7F8M1_DANRE        VSCGTWVGYQ FPGYRGSQYL LEKGEYRHFN EYGARCPQFQ SVRRIRDMQW HPHGCYTMAS K--------- ---------- ---------- ----------
CRBB1_HUMAN         VSSGTWVGYQ YPGYRGYQYL LEPGDFRHWN EWGAFQPQMQ SLRRLRDKQW HLEGSFPVLA TEPPK----- ---------- ---------- ----------
CRBB1_BOVIN         VSSGTWVGYQ YPGYRGYQYL LEPGDFRHWN EWGAFQPQMQ AVRRLRDRQW HREGCFPVLA AEPPK----- ---------- ---------- ----------
CRBB1_MOUSE         VSGGTWVGYQ YPGYRGYQYL LEPGDFRHWN EWGAFQPQMQ AVRRLRDRQW HQEGCFPVLT AEPPK----- ---------- ---------- ----------
Q6DGZ8_DANRE        VPGGAWVGYQ YPGYRGYQYL FECGDYRHYN EFCAFQPQIQ SVRRVRDMQF HQRGCFNMTA AKQ------- ---------- ---------- ----------
H3AS10_LATCH        VPCGTWVGYQ YPGYRGYQYL FECGDYRHWN EWCAFQPQMQ SMRRIRDMQW HQKGCFTFAS K--------- ---------- ---------- ----------
CRBB1_CHICK         VPSGTWVGYQ YPGYRGYQYL FETGDFRHWN EWCAFQPQIQ SIRRIRDMQW DQKGTFVTPE APSN------ ---------- ---------- ----------
A0A151MUJ3_ALLMI    VSSGTWVGYQ YPGYRGYQYL FERGDFRHWN EWSAFQPQIQ SIRRIRDLHW DRKGTYDTPE APSN------ ---------- ---------- ----------
```

Figure S9. Sequence alignment for β-crystallin Cluster 3 (βB1- and βB3-crystallins). Q52JI3_DANRE has an additional 7 amino acids (SATAASS) that were truncated to limit the figure to one page.

```
Q52JI4_DANRE        MATDHQNPAT KQKQPVASAF KLVIYEQENF QGRCHELTGP CNNLQEAGVE KVGSVLVQCG PWVGFEQPGC KGEQYVFEKG EYPRWDSWTN SRRSDCIVAF
CRBB2_CHICK         MASEHQMPAS KQQP---ASP NIAIFEQENF QGRCHELSGA CPNLKDAGVD KVGSILVHSG PWVGYEQASC KGEQFVFEKG EYPRWDSWTN SRRSDSITSL
A0A151NSD4_ALLMI    MASEHHLPLS K-QQ---TNP KIAIFEQENF QGRCHEINGS CTSLKEAGVE KVGSILVHSG PWVGYEQANC KGEQFVFEKG EYPRWDAWTN SRRSDIIGSL
H3AY34_LATCH        MASDHQAPAS KQQQ---ISS KIVLFEQENF QGRCHELSGP CSNLKEAGIE KVGSILVHCG PWVGYEQPNC KGEQYVFEKG EYPRWDSWTN SRRSDSISSL
F7A5Y5_ORNAN        ---------- ---------- -ITVFEQENF QGRSHELSGP CSNLKETGME KVASVLVHSG PWVGYEQTNC KGEQFVFEKG EYPRWDSWTN SRRSDCIGSL
B2ZF66_MACFL        MASDYQTQSS KPQP---LNP KIVIFEQENF QGRSHELSGP CSNLKETGME KAASILVQSG PWVGYEQANC KGEQFVFEKG EYPRWDSWTN SRRTDALSSL
CRBB2_MOUSE         MASDHQTQAG KPQP---LNP KIIIFEQENF QGHSHELSGP CPNLKETGME KAGSVLVQAG PWVGYEQANC KGEQFVFEKG EYPRWDSWTS SRRTDSLSSL
CRBB2_HUMAN         MASDHQTQAG KPQS---LNP KIIIFEQENF QGHSHELNGP CPNLKETGVE KAGSVLVQAG PWVGYEQANC KGEQFVFEKG EYPRWDSWTS SRRTDSLSSL
CRBB2_BOVIN         MASDHQTQAG KPQP---LNP KIIIFEQENF QGHSHELNGP CPNLKETGVE KAGSVLVQAG PWVGYEQANC KGEQFVFEKG EYPRWDSWTS SRRTDSLSSL

Q52JI4_DANRE        RPIKV----- ---------D SQEHKIVLYE NPSFTGKKIE IIDDDVPSFH AHGYHEKVSS VRVQSGTWVG YQYPGYRGFQ YLFEKGEFKE CSEFGAALPQ
CRBB2_CHICK         RPIKVVRAPR QPLPTRQTKD SQEHKIVLYE NPSFTGKKIE IIDDDVPSFH AHGYQEKVSS VRVQSGTWVG YQYPGYRGYQ YLFEKGDYKD SSEFGAQHPQ
A0A151NSD4_ALLMI    RPIKV----- ---------D SQEHKIVLFE NPSFTGKKIE IIDDDVPSFH AHGYQEKVSS VRVQSGTWVG YQYPGYRGYQ YLFEKGEYKD SSEFGAQHPQ
H3AY34_LATCH        RIIKV----- ---------D SQEHKIVLYE NPNFTGKKIE IIDDDVPSFH AHGYQEKVSS VRVQSGTWVG YQYPGYRGYQ YLFEKGDYKD SSEFGAQHPQ
F7A5Y5_ORNAN        RPVKV----- ---------D SQEHKIVLFE NPNFTGKKIE IIDDDVPSFH AHGYQEKVSS VRVQSGTWVG YQYPGYRGFQ YLFEKGEYKD SAEFGAQHPQ
B2ZF66_MACFL        RPIKV----- ---------D SQEHKIILYE NPNFTGKKIE IIDDDVPSFH AHGYQEKVSS VRVQSGTWVG YQYPGYRGFQ YLFEKGEYKD SSDFGAPHPQ
CRBB2_MOUSE         RPIKV----- ---------D SQEHKIILYE NPNFTGKKME IVDDDVPSFH AHGYQEKVSS VRVQSGTWVG YQYPGYRGLQ YLLEKGDYKD NSDFGAPHPQ
CRBB2_HUMAN         RPIKV----- ---------D SQEHKIILYE NPNFTGKKME IIDDDVPSFH AHGYQEKVSS VRVQSGTWVG YQYPGYRGLQ YLLEKGDYKD SSDFGAPHPQ
CRBB2_BOVIN         RPIKV----- ---------D SQEHKITLYE NPNFTGKKME VIDDDVPSFH AHGYQEKVSS VRVQSGTWVG YQYPGYRGLQ YLLEKGDYKD SGDFGAPQPQ

Q52JI4_DANRE        IQSVRRIRDM QYHPRGAFQA TS
CRBB2_CHICK         IQSVRRIRDM QWHQRGAYHP SN
A0A151NSD4_ALLMI    IQSVRRIRDM QWHQRGAFHP TN
H3AY34_LATCH        IQSVRRIRDM QWHQRGAFHP TN
F7A5Y5_ORNAN        IQSVRRIRDM QWHQRGAFHP AN
B2ZF66_MACFL        VQSVRRIRDM QWHQRGAFHP TN
CRBB2_MOUSE         VQSVRRIRDM QWHQRGAFHP SS
CRBB2_HUMAN         VQSVRRIRDM QWHQRGAFHP SN
CRBB2_BOVIN         VQSVRRIRDM QWHQRGAFHP SS
```

Figure S10. Sequence alignment for β-crystallin Cluster 4 (βB2-crystallins).
