# Supplementary material for: The Functional Significance of High Cysteine Content in Eye Lens γ-Crystallins
Source: Biomolecules. 2024 May 17;14(5):594. doi: 10.3390/biom14050594 (PMC11118217; doi:10.3390/biom14050594)
Supplement: Supplementary file 1 [file biomolecules-14-00594-s001.zip › D-I-Tasser_output.zip!/d_iTasser_output/Q5XTN6_DANRE_results/index.html]

D-I-TASSER DIT4782


[Back to server]
  

# D-I-TASSER results for DIT4782

#### [Click result.zip to download all results on this page]

**Input Sequence in FASTA format**

>Q5XTN6\_DANRE (178 residues)  
MGKVIFYEDRNFQGRNYECMSDCSDISSYLGRVGSIRVESGCFIVYERNGFMGNQFFLRR  
GEYHDIQRMMSMGMMFDTIRSCRMIPSYRGSFRMRIYERDNFGGQMYELMDDCESIQERY  
RMSDCQSCHVMDGHWLMFEQPHYRGRMIYFRPGEYRSFRDMGFSNMRFISMRRITDMC

  

**Predicted Secondary Structure**

**Sequence**

 20                  40                  60                  80                 100                 120                 140                 160                     \|                   \|                   \|                   \|                   \|                   \|                   \|                   \|  MGKVIFYEDRNFQGRNYECMSDCSDISSYLGRVGSIRVESGCFIVYERNGFMGNQFFLRRGEYHDIQRMMSMGMMFDTIRSCRMIPSYRGSFRMRIYERDNFGGQMYELMDDCESIQERYRMSDCQSCHVMDGHWLMFEQPHYRGRMIYFRPGEYRSFRDMGFSNMRFISMRRITDMC || **Prediction** |
 CCSSSSSSCCCCCSSSSSSCCCCCCCHHHCCCCCSSSSSCCCSSSSSCCCCCCSSSSSCCCCCCCHHHHCCCCCCCCCSSSSSSCCCCCCCCSSSSSSCCCCCCCSSSSCCCCCCHHHCCCCCCCCSSSSCCCCSSSSCCCCCCSSSSSSCCCCCCCHHHHCCCCCCSSSSSSCCCCC || **Confidence** | 9779998379981237994688876111117301299926127998389997348997588788857733689988833464202577887369999579998506998257987688167887437998269488970799833689975776799788278998145677633579 |
|  | H:Helix; S:Strand; C:Coil ||


**Predicted Solvent Accessibility**

|  |  |
| --- | --- |
| **Sequence** | 20                  40                  60                  80                 100                 120                 140                 160                     \|                   \|                   \|                   \|                   \|                   \|                   \|                   \|  MGKVIFYEDRNFQGRNYECMSDCSDISSYLGRVGSIRVESGCFIVYERNGFMGNQFFLRRGEYHDIQRMMSMGMMFDTIRSCRMIPSYRGSFRMRIYERDNFGGQMYELMDDCESIQERYRMSDCQSCHVMDGHWLMFEQPHYRGRMIYFRPGEYRSFRDMGFSNMRFISMRRITDMC |
| **Prediction** | 7320100036605123221444134035205301002035431000133614021000341414324302442433210201322434444120101137615333220343142046333344020020351320012245140210103425143254122533301002313768 |
|  | Values range from **0** (buried residue) to **8** (highly exposed residue) ||

**Predicted Contact and Distance Map Used in D-I-TASSER simulation**

|  |  |  |  |  |  |  |  |  |
| --- | --- | --- | --- | --- | --- | --- | --- | --- |
| **Contact Map** \| **Distance Map** \| **Hydrogen bond networks** | | | | | | | | |

|  |
| --- |
| D-I-TASSER simulation is guided by the consensus contact map (left figure), distance map (middle figure) and Hydrogen bond network (right figure) derived based on confidence scores of AttentionPotential. In the contact, distance map and hydrogen bond networks, the axes mark the residue index along the sequence. For the contact map, each dot represents a residue pair with predicted contact, while for the distance map and hydrogen bond network, a color scale represents a distance of 1-20+ angstroms or a angle of 0-180 degree. |


**Top 10 threading templates used by D-I-TASSER**

|  |  |  |  |  |  |  |  |  |
| --- | --- | --- | --- | --- | --- | --- | --- | --- |
| Rank | PDB | ID1 | ID2 | Cov | Norm. | Downloadalignment |  | 20                  40                  60                  80                 100                 120                 140                 160                     \|                   \|                   \|                   \|                   \|                   \|                   \|                   \| |
|  | | | | | | | SS | CCSSSSSSCCCCCSSSSSSCCCCCCCHHHCCCCCSSSSSCCCSSSSSCCCCCCSSSSSCCCCCCCHHHHCCCCCCCCCSSSSSSCCCCCCCCSSSSSSCCCCCCCSSSSCCCCCCHHHCCCCCCCCSSSSCCCCSSSSCCCCCCSSSSSSCCCCCCCHHHHCCCCCCSSSSSSCCCCC |
| 1 | 1zwmA | 0.44 | 0.42 | 12.34 | **1.33** | DEthreader |  | GGKISFYEDRNFQGRRYDCDCDCADFRSYLSRCNSIRVEGGTWAVYERPFSGHMY-ILPQGEYPEYQRW--M-GLNDRLGSCRAVHLSSGQAKIQVFEKGDFNGQMYETTEDCPSIMEQFHLREIHSCKVVEGTWIFYELPNYRGRQYLLDKKEYRKPVDWGAASPAIQSFRRIV-E- |
| 2 | 1zwmA | 0.42 | 0.41 | 12.04 | **1.17** | DEthreader |  | GGKISFYEDRNFQGRRYDCDCDCADFRSYLSRCNSIRVEGGTWAVYERPNFSGHMYILPQGEYPEY-QRWMGLN-D-RLGSCRAVHLSSGQAKIQVFEKGDFNGQMYETTEDCPSIMEQFHLREIHSCKVVEGTWIFYELPNYRGRQYLLDKKEYRKPVDGAASPA-IQSFRRIV--E |
| 3 | 2jdfA | 0.52 | 0.51 | 14.63 | **3.73** | SPARKS-K |  | -GKITFYEDRAFQGRSYECTTDCPNLQPYFSRCNSIRVESGCWMIYERPNYQGHQYFLRRGEYPDYQQWMGL---SDSIRSCCLIPPHSGAYRMKIYDRDELRGQMSELTDDCLSVQDRFHLTEIHSLNVLEGSWILYEMPNYRGRQYLLRPGEYRRFLDWGAPNAKVGSLRRVMDLY |
| 4 | 1blbC | 0.30 | 0.30 | 9.03 | 0.90 | CEthreader |  | NPKIIIFEQENFQGHSHELNGPCPNLKETVEKAGSVLVQAGPWVGYEQANCKGEQFVFEKGEYPRWDSWTS-SRRTDSLSSLRPIKVDSQEHKITLYENPNFTGKKMEVIDDVPSFHAHGYQEKVSSVRVQSGTWVGYQYPGYRGLQYLLEKGDYKDSGDFGAPQPQVQSVRRIRDMQ |
| 5 | 2jdfA | 0.52 | 0.51 | 14.63 | **3.27** | MUSTER |  | -GKITFYEDRAFQGRSYECTTDCPNLQPYFSRCNSIRVESGCWMIYERPNYQGHQYFLRRGEYPDYQQWMGL---SDSIRSCCLIPPHSGAYRMKIYDRDELRGQMSELTDDCLSVQDRFHLTEIHSLNVLEGSWILYEMPNYRGRQYLLRPGEYRRFLDWGAPNAKVGSLRRVMDLY |
| 6 | 2m3cA | 0.64 | 0.62 | 17.81 | **2.87** | HHsearch |  | MGKIIFYEDRNFGGRYHECMSDCADLHSYFNRCHSIRVESGCFMVYDRTNFMGRQYFLRRGEYPDYMRTMGM---NDCVRSCRMIPLHHGSFKMRLYEHSDMGGRMMELMDDCPNLMDRFNMSDFHSCHVMDGHWLVYEQPNYTGRQFYLRPGEYRSYNDWGGVTSRMGSIRRITDL- |
| 7 | 2jdfA | 0.50 | 0.49 | 14.17 | **2.46** | FFAS-3D |  | -GKITFYEDRAFQGRSYECTTDCPNLQPYFSRCNSIRVESGCWMIYERPNYQGHQYFLRRGEYPDYQQWMGLSDSIR---SCCLIPPHSGAYRMKIYDRDELRGQMSELTDDCLSVQDRFHLTEIHSLNVLEGSWILYEMPNYRGRQYLLRPGEYRRFLDWGAPNAKVGSLRRVMDL- |
| 8 | 1i5iA | 0.52 | 0.51 | 14.62 | **3.85** | CNFpred |  | -GKITFYEDRGFQGHCYESSSDCPNLQPYFSRCNSIRVDSGCWMLYERPNYQGHQYFLRRGDYPDYQQWMGF---NDSIRSCRLIPQHTGTFRMRIYERDDFRGQMSEITDDCPSLQDRFHLTEVHSLNVLEGSWVLYEMPSYRGRQYLLRPGEYRRYLDWGAMNAKVGSLRRVMDF- |
| 9 | 2jdfA | 0.46 | 0.44 | 12.95 | **1.17** | DEthreader |  | -GKITFYEDRAFQGRSYECTTDCPNLQPYFSRCNSIRVESGCWMIY-ERPQGHQYFLRR-G-E-YPD-YQQWMGLSDSIRSCCLIPPHSGAYRMKIYDRDELRGQMSELTDDCLSVQDRFHLTEIHSLNVLEGSWILYEMPNYRGRQYLLRPGEYRRFLDWGAPNAKVGSLRRVMDLY |
| 10 | 2m3cA | 0.61 | 0.60 | 17.05 | **1.17** | DEthreader |  | MGKIIFYEDRNFGGRYHECMSDCADLHSYFNRCHSIRVESGCFMVYDRTNFMGRQYFLRRGEY-P--DYMRTGMND-CVRSCRMIPLHHGSFKMRLYEHSDMGGRMMELMDDCPNLMDRFNMSDFHSCHVMDGHWLVYEQPNYTGRQFYLRPGEYRSYNDWGGVTSRMGSIRR-ITDL |
| (a) \| *ID1* is the number of template residues identical to query divided by number of aligned residues.; (b) \| *ID2* is the number of template residues identical to query divided by query sequence length.; (c) \| *Cov* is equal the number of aligned template residues divided by query sequence length.; (d) \| *Norm. Zscore* is the normalized Z-score of the threading alignments. A Normalized Z-score >1 means a good alignment and is highlighted in **bold**.; (e) \| *Download alignment* lists the threading program used to identify the template, and provide the 3D structure of aligned regions of threading templates (threading[1-10].pdb.gz).; (f) \| Template residues identical to query sequence are highlighted in color. | | | | | | | | |

**Top 2 final models from D-I-TASSER**

  

| Click to view \| Rank^a^ \| Download PDB \| Download ModelCIF \| Estimated TM-score^b^; \| 1 \| model1.pdb.gz \| model1.cif \| 0.84 \|; \| 2 \| model2.pdb.gz \| \| 0.52 (a) \| D-I-TASSER simulations generate a large ensemble of structural conformations, i.e. decoys. These decoys are clustered by SPICKER based on pairwise structure similarity to report up to five final models from the five largest clusters. Models are ranked in descending order of cluster size. If the simulations converge well, it is possible to have less than 5 models generated, which is usually an indication of good model quality.; (b) \| The model confidence is quantitatified by estimated TM-score (eTM-score), calculated based on significance of threading template alignments, contact map satisfaction rate, mean absolute error between distance of model and distance of AttentionPotential, and convergence of D-I-TASSER simulations. eTM-score is typically in the range of [0, 1], with higher eTM-score signifies higher model confidence. |


#### [Click result.zip to download all results on this page]

---
