# Supplementary material for: The Functional Significance of High Cysteine Content in Eye Lens γ-Crystallins
Source: Biomolecules. 2024 May 17;14(5):594. doi: 10.3390/biom14050594 (PMC11118217; doi:10.3390/biom14050594)
Supplement: Supplementary file 1 [file biomolecules-14-00594-s001.zip › D-I-Tasser_output.zip!/d_iTasser_output/A0A8C4N311_EPTBU_results/index.html]

D-I-TASSER DIT4641


[Back to server]
  

# D-I-TASSER results for DIT4641

#### [Click result.zip to download all results on this page]

**Input Sequence in FASTA format**

>A0A8C4N311\_EPTBU (153 residues)  
KIQVVLYEEEEFCGRSQEMCVTLAIYLFVCSSWVIYEEPGFQGRQLLLEEGEFPRPSAWG  
GYSGRLGSIRPICCVSSTFHNHLLSSTRVVPEHYLEGCRWVAYEEANFCGEQYILERGLY  
RSHLDWGGSDPSIGSVRPVFLVRLPMTMKLHFI

  

**Predicted Secondary Structure**

**Sequence**

 20                  40                  60                  80                 100                 120                 140                     \|                   \|                   \|                   \|                   \|                   \|                   \|  KIQVVLYEEEEFCGRSQEMCVTLAIYLFVCSSWVIYEEPGFQGRQLLLEEGEFPRPSAWGGYSGRLGSIRPICCVSSTFHNHLLSSTRVVPEHYLEGCRWVAYEEANFCGEQYILERGLYRSHLDWGGSDPSIGSVRPVFLVRLPMTMKLHFI || **Prediction** |
 CCSSSSSSCCCCCCCSSSCCCCHHHHSSSCCCSSSSSCCCCCCSSSSSCCCCCCCHHHCCCCCCCSSSSSSSSCCCCCCCCSSSSSCCCCCCSSSSCCCSSSSSCCCCCSSSSSSCCCCCCCHHHHCCCCCCSSSSSSSSCCCCCCCCCSSSC || **Confidence** | 968999957998540787089913411054857999279992047886588789844436789961556778335888630478634776631881561999828998036999758986997883789980465788641678764331429 |
|  | H:Helix; S:Strand; C:Coil ||


**Predicted Solvent Accessibility**

|  |  |
| --- | --- |
| **Sequence** | 20                  40                  60                  80                 100                 120                 140                     \|                   \|                   \|                   \|                   \|                   \|                   \|  KIQVVLYEEEEFCGRSQEMCVTLAIYLFVCSSWVIYEEPGFQGRQLLLEEGEFPRPSAWGGYSGRLGSIRPICCVSSTFHNHLLSSTRVVPEHYLEGCRWVAYEEANFCGEQYILERGLYRSHLDWGGSDPSIGSVRPVFLVRLPMTMKLHFI |
| **Prediction** | 822010014660432323114404322332100000133714221120353513324302433330100210324375241311324541221304412000012461422110034361431431226333010022031463445140114 |
|  | Values range from **0** (buried residue) to **8** (highly exposed residue) ||

**Predicted Contact and Distance Map Used in D-I-TASSER simulation**

|  |  |  |  |  |  |  |  |  |
| --- | --- | --- | --- | --- | --- | --- | --- | --- |
| **Contact Map** \| **Distance Map** \| **Hydrogen bond networks** | | | | | | | | |

|  |
| --- |
| D-I-TASSER simulation is guided by the consensus contact map (left figure), distance map (middle figure) and Hydrogen bond network (right figure) derived based on confidence scores of AttentionPotential. In the contact, distance map and hydrogen bond networks, the axes mark the residue index along the sequence. For the contact map, each dot represents a residue pair with predicted contact, while for the distance map and hydrogen bond network, a color scale represents a distance of 1-20+ angstroms or a angle of 0-180 degree. |


**Top 10 threading templates used by D-I-TASSER**

|  |  |  |  |  |  |  |  |  |
| --- | --- | --- | --- | --- | --- | --- | --- | --- |
| Rank | PDB | ID1 | ID2 | Cov | Norm. | Downloadalignment |  | 20                  40                  60                  80                 100                 120                 140                     \|                   \|                   \|                   \|                   \|                   \|                   \| |
|  | | | | | | | SS | CCSSSSSSCCCCCCCSSSCCCCHHHHSSSCCCSSSSSCCCCCCSSSSSCCCCCCCHHHCCCCCCCSSSSSSSSCCCCCCCCSSSSSCCCCCCSSSSCCCSSSSSCCCCCSSSSSSCCCCCCCHHHHCCCCCCSSSSSSSSCCCCCCCCCSSSC |
| 1 | 1zq7B | 0.08 | 0.07 | 2.63 | 0.83 | DEthreader |  | TAKLKTIFLSGKSP---SGVE-LSPVFEYRGVFVTLTEGGL-LRGCIGHALDSISATRDPRFPTVEILVEVTI-L--TQPEKINASKEPDKVEIGK-HGLIVK-QGYCQ--GLLLPQADSIDF-LSCPDAVKGAEVYC-FEGQIFK---P--- |
| 2 | 1sddB | 0.06 | 0.05 | 2.24 | 0.83 | DEthreader |  | QIQAS--LST-EFNPEPWIQVDMQKEV-LLTGIQTQGAKHYASTIKENQIDPISPTG---S-YNKPALRLELQGCEVNGSTPLMESGKILKKKIT---AIVTQGCKSEMYVKYFNFPIISRF-IRIIPKSIALRLELF--------------- |
| 3 | 2jdfA | 0.34 | 0.31 | 9.39 | **3.03** | SPARKS-K |  | -GKITFYEDRAFQGRSYECTTD--CPNLNSGCWMIYERPNYQGHQYFLRRGEYPDYQQWMGLSDSIRSCCLIPPHSGAYRMKIYDRDDDCLSNVLEGS-WILYEMPNYRGRQYLLRPGEYRRFLDWGAPNAKVGSLRRVMDLYL--------- |
| 4 | 1blbC | 0.30 | 0.28 | 8.53 | 0.74 | CEthreader |  | NPKIIIFEQENFQGHSHELNGPCGSVLVQAGPWVGYEQANCKGEQFVFEKGEYPRWDSWTSSTDSLSSLRPIKVDSQEHKITLYENQEKVSSVRVQSGTWVGYQYPGYRGLQYLLEKGDYKDSGDFGAPQPQVQSVRRIRDMQW--------- |
| 5 | 2jdfA | 0.32 | 0.30 | 9.05 | **2.20** | MUSTER |  | -GKITFYEDRAFQGRSYECTTDCPNLQVESGCWMIYERPNYQGHQYFLRRGEYPDYQQWMGLSDSIRSCCLIPPHSGAYRMKIYDRTDDCLSLNVLEGSWILYEMPNYRGRQYLLRPGEYRRFLDWGAPNAKVGSLRRVMDLYL--------- |
| 6 | 2jdfA | 0.35 | 0.32 | 9.56 | **2.67** | HHsearch |  | -GKITFYEDRAFQGRSYECTTD--CPNLQSGCWMIYERPNYQGHQYFLRRGEYPDYQQWMGLSDSIRSCCLIPPHSGAYRMKIYDRTDDCLSNVLEG-SWILYEMPNYRGRQYLLRPGEYRRFLDWGAPNAKVGSLRRVMDLYL--------- |
| 7 | 2jdfA | 0.31 | 0.29 | 8.68 | **1.75** | FFAS-3D |  | --KITFYEDRAFQGRSYECTTDCNSIRVESGCWMIYERPNYQGHQYFLRRGEYPDYQQWMGLSDSIRSCCLIPPHSGAYRMKIYDHLTEIHSLNVLEGSWILYEMPNYRGRQYLLRPGEYRRFLDWGAPNAKVGSLRRVMDL----------- |
| 8 | 5vh1A | 0.31 | 0.28 | 8.50 | **2.46** | CNFpred |  | GPKVTFYEDKNFLGRRYECDA-CNSIRVEGGTWVAYERPNYSGNMYVLRRGEYPDYHHWMGLNDRLGSCKAVHIPGAQGHIQVFEKGREVHACRVLEGIWVFYEHPNYRGRQYLLPKGEYRQPVEWGAVTPAVQSFRSIAE------------ |
| 9 | 1wscA | 0.07 | 0.06 | 2.45 | 0.83 | DEthreader |  | NVKIDSIAN--KLGILKI-----PILNKKGLAFVTLETYYSTSLGCIGYIVKAIAAFSDPRFPPLFIIIEVTV-L--T-PQEIDVRWELPKKIKVGEDGLIVEYG---ILYSGLLLPPEETFLAETCGLECNKVKIKK-FQGIIFR---P--- |
| 10 | 4d8oA | 0.05 | 0.04 | 1.88 | 0.83 | DEthreader |  | -----GLRIIIVIVEIPHFAA--LRGKE--RELVVLRSNGDSWKEHFCD-PLCRIITR---DFPQYFAVVSRKQTVVPQVQALERFHKPITMTIPVPKLLCSITGGTTPAQWEDITCVSFTTN---------V-SARFWYEICVPYMAD---- |
| (a) \| *ID1* is the number of template residues identical to query divided by number of aligned residues.; (b) \| *ID2* is the number of template residues identical to query divided by query sequence length.; (c) \| *Cov* is equal the number of aligned template residues divided by query sequence length.; (d) \| *Norm. Zscore* is the normalized Z-score of the threading alignments. A Normalized Z-score >1 means a good alignment and is highlighted in **bold**.; (e) \| *Download alignment* lists the threading program used to identify the template, and provide the 3D structure of aligned regions of threading templates (threading[1-10].pdb.gz).; (f) \| Template residues identical to query sequence are highlighted in color. | | | | | | | | |

**Top 5 final models from D-I-TASSER**

  

| Click to view \| Rank^a^ \| Download PDB \| Download ModelCIF \| Estimated TM-score^b^; \| 1 \| model1.pdb.gz \| model1.cif \| 0.65 \|; \| 2 \| model2.pdb.gz \| \| 0.62; \| 3 \| model3.pdb.gz \| \| 0.49; \| 4 \| model4.pdb.gz \| \| 0.48; \| 5 \| model5.pdb.gz \| \| 0.48 (a) \| D-I-TASSER simulations generate a large ensemble of structural conformations, i.e. decoys. These decoys are clustered by SPICKER based on pairwise structure similarity to report up to five final models from the five largest clusters. Models are ranked in descending order of cluster size. If the simulations converge well, it is possible to have less than 5 models generated, which is usually an indication of good model quality.; (b) \| The model confidence is quantitatified by estimated TM-score (eTM-score), calculated based on significance of threading template alignments, contact map satisfaction rate, mean absolute error between distance of model and distance of AttentionPotential, and convergence of D-I-TASSER simulations. eTM-score is typically in the range of [0, 1], with higher eTM-score signifies higher model confidence. |


**Proteins with similar structure**

**Top 10 structural analogs in PDB (as identified by
TM-align)**

**Predicted Gene Ontology (GO) Terms**

**Molecular Function (MF)**

|  |  |  |
| --- | --- | --- |
| **GO term** | **Cscore^GO^** | **Name** |
| GO:0005198 | 0.87 | structural molecule activity |
| GO:0005212 | 0.85 | structural constituent of eye lens |

  

Download full result of the above consensus prediction.

  

|  |  |
| --- | --- |
|  | **Click the graph to show a high resolution version.** |
| (a) | Cscore^GO^ is the confidence score of predicted GO terms. Cscore^GO^ values range in between [0-1]; where a higher value indicates a better confidence in predicting the function using the template. |
| (b) | The graph shows the predicted terms within the Gene Ontology hierachy for Molecular Function. Confidently predicted terms are color coded by Cscore^GO^: |
|  | [0.4,0.5) \| [0.5,0.6) \| [0.6,0.7) \| [0.7,0.8) \| [0.8,0.9) \| [0.9,1.0] |


**Biological Process (BP)**

|  |  |  |
| --- | --- | --- |
| **GO term** | **Cscore^GO^** | **Name** |
| GO:0044767 | 0.80 | single-organism developmental process |
| GO:0048856 | 0.74 | anatomical structure development |
| GO:0007601 | 0.68 | visual perception |
| GO:0002088 | 0.50 | lens development in camera-type eye |

  

Download full result of the above consensus prediction.

  

|  |  |
| --- | --- |
|  | **Click the graph to show a high resolution version.** |
| (a) | Cscore^GO^ is the confidence score of predicted GO terms. Cscore^GO^ values range in between [0-1]; where a higher value indicates a better confidence in predicting the function using the template. |
| (b) | The graph shows the predicted terms within the Gene Ontology hierachy for Biological Process. Confidently predicted terms are color coded by Cscore^GO^: |
|  | [0.4,0.5) \| [0.5,0.6) \| [0.6,0.7) \| [0.7,0.8) \| [0.8,0.9) \| [0.9,1.0] |


**Cellular Component (CC)**

|  |  |  |
| --- | --- | --- |
| **GO term** | **Cscore^GO^** | **Name** |
| GO:0005737 | 1.00 | cytoplasm |
| GO:0005634 | 1.00 | nucleus |

  

Download full result of the above consensus prediction.

  

|  |  |
| --- | --- |
|  | **Click the graph to show a high resolution version.** |
| (a) | Cscore^GO^ is the confidence score of predicted GO terms. Cscore^GO^ values range in between [0-1]; where a higher value indicates a better confidence in predicting the function using the template. |
| (b) | The graph shows the predicted terms within the Gene Ontology hierachy for Cellular Component. Confidently predicted terms are color coded by Cscore^GO^: |
|  | [0.4,0.5) \| [0.5,0.6) \| [0.6,0.7) \| [0.7,0.8) \| [0.8,0.9) \| [0.9,1.0] |

**Predicted Enzyme Commission (EC) Numbers**

**Top 5 enzyme homologs in PDB**

**Predicted Ligand Binding Sites**

**Template proteins with similar binding site:**

#### [Click result.zip to download all results on this page]

---
